# Distinct and Site-Specific Phosphorylation of the Retinoblastoma Protein at Serine 612 in Differentiated Cells

**DOI:** 10.1371/journal.pone.0086709

**Published:** 2014-01-21

**Authors:** Takayuki Hattori, Chiharu Uchida, Hirotaka Takahashi, Naoki Yamamoto, Mikihiko Naito, Yoichi Taya

**Affiliations:** 1 Division of Biochemistry and Molecular Biology, National Institute of Health Sciences, Setagaya-ku, Tokyo, Japan; 2 Cancer Science Institute of Singapore, National University of Singapore, Singapore, Singapore; 3 Research Equipment Center, Hamamatsu University School of Medicine, Hamamatsu, Shizuoka, Japan; 4 Division of Cell-Free Sciences, Proteo-Science Center, Ehime University, Matsuyama, Ehime, Japan; 5 Department of Microbiology, Yong Loo Lin School of Medicine, National University of Singapore, Singapore, Singapore; 6 Department of Biochemistry, National University of Singapore, Singapore, Singapore; National Cancer Center Research Institute, Japan

## Abstract

The retinoblastoma susceptibility protein (pRB) is a phosphoprotein that regulates cell cycle progression at the G1/S transition. In quiescent and early G1 cells, pRB predominantly exists in the active hypophosphorylated form. The cyclin/cyclin-dependent protein kinase complexes phosphorylate pRB at the late G1 phase to inactivate pRB. This event leads to the dissociation and activation of E2F family transcriptional factors. At least 12 serine/threonine residues in pRB are phosphorylated *in vivo*. Although there have been many reports describing bulk phosphorylation of pRB, detail research describing the function of each phosphorylation site remains unknown. Besides its G1/S inhibitory function, pRB is involved in differentiation, prevention of cell death and control of tissue fate. To uncover the function of phosphorylation of pRB in various cellular conditions, we have been investigating phosphorylation of each serine/threonine residue in pRB with site-specific phospho-serine/threonine antibodies. Here we demonstrate that pRB is specifically phosphorylated at Ser612 in differentiated cells in a known kinase-independent manner. We also found that pRB phosphorylated at Ser612 still associates with E2F-1 and tightly binds to nuclear structures including chromatin. Moreover, expression of the Ser612Ala mutant pRB failed to induce differentiation. The findings suggest that phosphorylation of Ser612 provides a distinct function that differs from the function of phosphorylation of other serine/threonine residues in pRB.

## Introduction

The retinoblastoma tumor suppressor protein (pRB) plays a pivotal role in regulating the cell cycle progression at the G1/S transition [1]. pRB exists predominantly in a hypophosphorylated form in quiescent and early G1 cells. Hypophosphorylated pRB binds to the transcriptional activation domain of E2Fs and represses their activity by recruiting histone deacetylase 1, resulting in the suppression of target genes required for cell cycle progression and S phase entry. Upon mitogenic stimulation, the cyclin-dependent protein kinases (Cdks) phosphorylate pRB to allow the precise inactivation of its function in the mid-to-late G1 phase, thereby facilitating the entry of cells into the S phase [2]. Inactivation of pRB by phosphorylation leads to the release of pRB from E2F, and therefore the expression of target genes regulated by E2Fs. Cdk4/6-cyclin D and Cdk2-cyclin E complexes sequentially inactivate pRB by phosphorylating pRB in the course of normal cell cycle progression [2]. There are 16 potential serine/threonine-proline phospho-acceptor sites for Cdk-mediated phosphorylation in pRB and 12 of them are phosphorylated *in vivo* [3], [4]. It has also been reported that some of these residues are phosphorylated by distinct Cdks [5]–[11].

Besides its cell cycle regulation, pRB is involved in cell death and differentiation via its ability to activate several transcription factors and to recruit SWI/SNF chromatin-remodeling factors [12]. It has been reported that hypophosphorylation of pRB is correlated with cell differentiation [13]. Since only hypophosphorylated pRB can bind to a viral oncoprotein [14] and can associate with the nuclear compartment [15], it has been suggested that phosphorylation of pRB is required to inactivate the protein-protein interacting ability of pRB. There have been many reports describing bulk phosphorylation of pRB; however, there is a paucity of information describing the role of each phosphorylation site. Therefore, we have been investigating phosphorylation of each serine/threonine residue by taking advantage of site-specific phospho-serine/threonine antibodies in various cellular conditions.

In this report, we show that phosphorylation of pRB at Ser612 is promoted during cell differentiation, whereas other serine/threonine residues are dephosphorylated. Distinct from phosphorylation of pRB at other serine/threonine residues, pRB phosphorylated at Ser612 remains associated with E2F-1. We also demonstrate that Ser612-phosphorylated pRB binds tightly to nuclear structures, including chromatin, whereas other serine/threonine residues in chromatin-bound pRB are dephosphorylated. Furthermore, the expression of the Ser612Ala mutant pRB markedly impaired neural differentiation in SH-SY5Y cells. These findings raise the question about the function of phosphorylation of individual Ser/Thr residues in pRB with respect to facilitating the dissociation of pRB with its binding partners and nuclear structures.

## Materials and Methods

### Reagents

RPMI1640 medium, DMEM, TPA, all-trans retinoic acid (ATRA), hemin, etoposide and GW8510 were purchased from Sigma (St. Louis, MO, USA). Chk2 inhibitor II, SB218078 and PD98059 were from Merck (Darmstadt, Germany). λ protein phosphatase was obtained from New England Biolabs (Ipswich, MA, USA). Anti-pRB monoclonal antibody (G3–245), anti-underphosphorylated (under-P) pRB monoclonal antibody (G99–549) and anti-Hsp90 monoclonal antibody (68) were from BD Biosciences (Franklin Lakes, NJ, USA). Anti-Phospho-Chk2 (Thr68) polyclonal antibody and anti-Myc monoclonal antibody (9B11) were from Cell Signaling Technology (Danvers, MA, USA). Anti-Lamin A/C monoclonal antibody (636), anti-Chk2 polyclonal antibody (H-300) and anti-E2F-1 polyclonal antibody (C-20) were from Santa Cruz (Santa Cruz, CA, USA). Phospho-specific pRB monoclonal (pThr356, pSer612 and pSer807) and polyclonal (pThr373, pSer608, pSer780, pSer795, pSer811, pThr821 and pThr826) antibodies were previously described [16]–[21].

### Cell Culture and Induction of Differentiation

All cell lines were from American Type Culture Collection (Manassas, VA, USA). U-937 cells, K562 cells and MOLT-4 were cultured in RPMI1640 medium containing 10% FBS and antibiotics. SAOS-2 cells, C33A cells and U2OS cells were grown in DMEM containing 10% FBS and antibiotics. SH-SY5Y cells were maintained in DMEM supplemented with 10% FBS, 0.1 mM non-essential amino acids, 1 mM sodium pyruvate and antibiotics. Cells were grown in 5% CO_2_ at 37°C in a humidified atmosphere. For induction of differentiation, U-937 cells and K562 cells at a concentration of 2×10^5^ cells/ml were cultured for 24 h and then incubated in complete medium with TPA or hemin for 96 h at the indicated concentration. SH-SY5Y cells were cultured in the presence of 10 µM of ATRA for 14 days to induce neural differentiation. After differentiation, cells were assessed by flow cytometric analysis of cell surface antigens and DNA contents and/or morphologic analysis.

### Immunoprecipitation and Immunoblotting

Cell lysates were prepared with TNT buffer (50 mM Tris-HCl, pH 7.5, 300 mM NaCl and 0.5% Triton-X 100) supplemented with protease inhibitors and phosphatase inhibitors and subjected to immunoprecipitation and immunoblotting as described previously with some modifications [22].

### Cloning of RB ORF into the pEU Vector and the Purification of Recombinant pRB

The pcDNA3.1 vector containing full-length *RB* with a 3×Flag-tag at the 5′ end was digested with *Xho*I and *Not*I, which are restriction sites located upstream and downstream of the ORF of *RB*, respectively. The fragment of the *RB* ORF was inserted into the pEU-E01 vector with a *GST-tag*, TEV protease recognition sequence and a biotin ligase recognition site upstream of the MCS. Using this plasmid as the template, *in vitro* transcription and bilayer cell-free protein synthesis were performed according to a published procedure [23] with slight modifications. The purification of the GST-tagged pRB was carried out using glutathione-conjugated magnet beads (Promega, Madison, WI, USA) according to the manufacturer’s procedure. Briefly, 250 µl of the translation mixture was mixed with the beads and incubated for 2 h at 4°C with gentle rotation. After removing the supernatant, bound GST-tagged pRB was washed four times with PBS (−), and the GST-tagged pRB protein was eluted with 50 µl of a reduced glutathione buffer.

### Mammalian Expression Vectors and Transfection

Expression vectors for wild-type pRB and mutant pRB in which the Ser612 residue was substituted with alanine (pRB S612A) and asparagine (pRB S612D) were constructed previously [18]. Cells were transfected with the FuGENE 6 transfection reagent (Roche, Indianapolis, IN, USA) according to the manufacturer’s protocol.

### Fractionation of Cellular Proteins

Cells were subjected to sequential extraction with detergent and salt according to a previously reported method with some modifications [24]. In brief, the cells were suspended in a hypotonic buffer (10 mM HEPES, pH 7.9, 10 mM KCl, 1.5 mM MgCl_2_) and lysed with 0.1% Triton X-100. The lysates were centrifuged to yield the clear supernatant CS1 (cytoplasmic soluble fraction). The pellet was washed twice with isotonic sucrose buffer (50 mM Tris-HCl, pH 7.4, 0.25 M sucrose, 5 mM MgCl_2_) to yield the CS2 fraction (cytoplasmic fraction). The nuclear envelope was removed by a low salt (LS) buffer (10 mM Tris-HCl, pH 7.4, 0.2 mM MgCl_2_) containing 1% Triton X-100 to yield the NS fraction (nucleoplasmic soluble fraction). The nuclear pellet was washed twice with LS buffer and extracted sequentially with 300 mM and 500 mM NaCl in the LS buffer to give the supernatant fractions, respectively designated as 0.3 and 0.5. The nuclear residue (NR) comprising DNA and the nuclear matrix was resuspended in LS buffer and solubilized by sonication. All buffers were supplemented with protease inhibitors and phosphatase inhibitors.

### Immunofluorescence

Cells were fixed with ice-cold methanol-acetone, blocked with 5% BSA and treated with an anti-Myc antibody followed by treatment with AlexaFluor488-conjugated goat anti-mouse IgG and DAPI. Fluorescent images were obtained using an IX73 microscope (Olympus, Tokyo, Japan).

### Establishment of pRB-inducible SAOS-2 Cell Clones and Senescence β-galactosidase Assay

We established pRB-inducible SAOS-2 cell clones by taking advantage of the T-REx System (Invitrogen, Carlsbad, CA, USA) according to the manufacturer’s protocol. Wild-type and Ser612Ala mutant pRB cDNAs containing the C-terminal FLAG epitope tag were cloned into pcDNA5/TO. Four clones (WT7 and WT21 for wild-type pRB clones and SA17 and SA27 for Ser612Ala) in which pRB expression was induced by doxycycline treatment in a dose-dependent manner were established (Fig. S1A). Osteogenic senescence-like differentiated cells were evaluated by flat cell formation and using the senescence β-galactosidase assay kit (Cell Signaling Technology).

## Results

### Sustained Phosphorylation of the Ser612 Residue in pRB in Differentiating Cells

**Figure 1:**
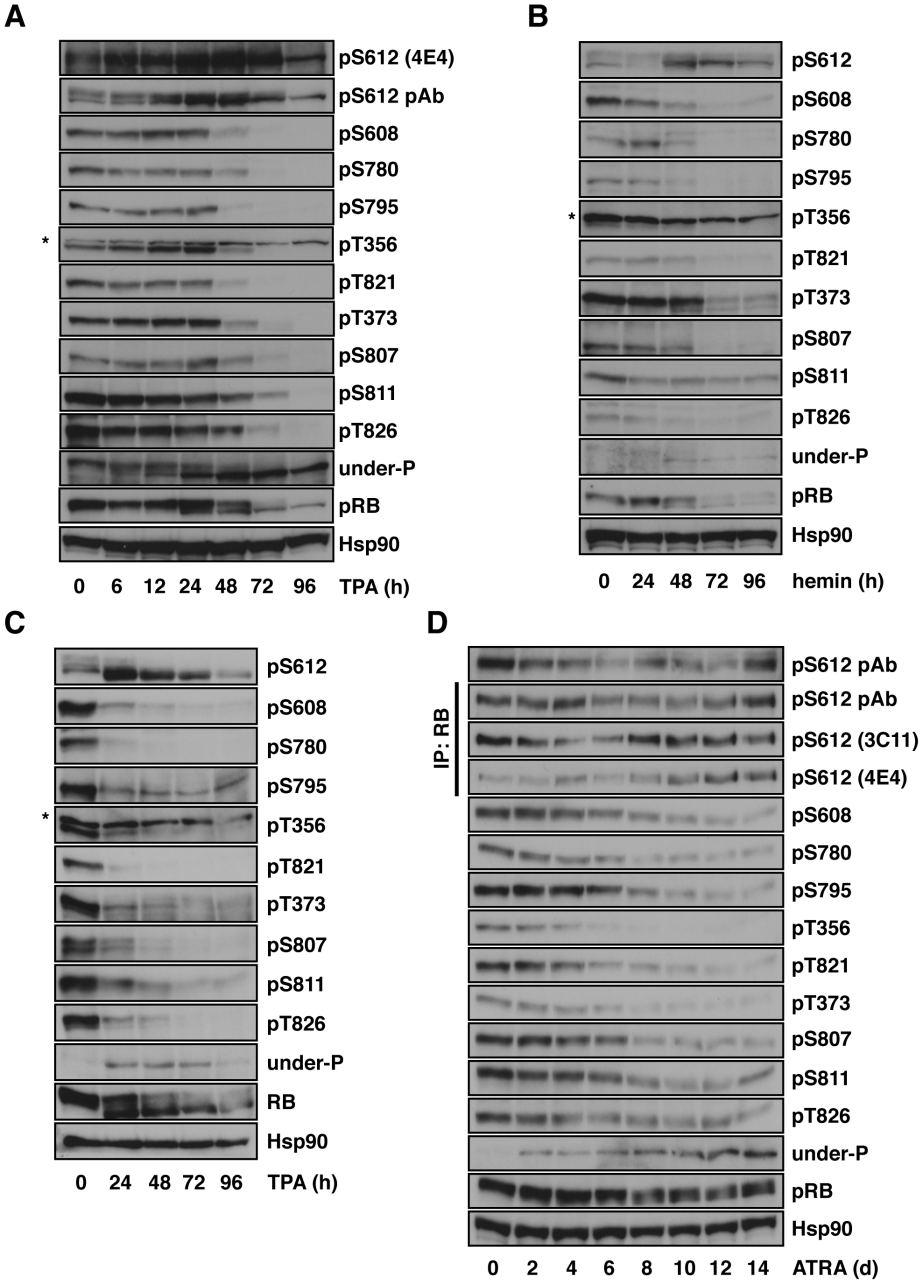
pRB is phosphorylated at Ser612 in differentiated cells. (A) U-937 cells were treated with 20 nM TPA for the indicated periods and then the cells were harvested for immunoblotting with the indicated antibodies. (B and C) K562 cells were treated with 30 µM hemin (B) or 10 nM TPA (C) for the indicated periods and then the cells were harvested for immunoblotting with the indicated antibodies. (D) SH-SY5Y cells were treated with 10 µM ATRA for the indicated periods and then the cells were harvested for immunoprecipitation and immunoblotting with the indicated antibodies. The asterisks denote non-specific bands.

pRB is a multifunctional protein. In addition to cell cycle control, pRB plays an essential role in cellular differentiation, senescence and apoptosis. Inactivation of pRB impairs differentiation in vitro and in vivo. pRB is a phosphoprotein with at least 12 serine/threonine residues that are phosphorylated in vivo [3], [4]. For these reasons, we hypothesized that the phosphorylation or dephosphorylation of particular serine or threonine residues is required for full induction of cell differentiation. We therefore examined changes in the phosphorylation status of serine/threonine residues in pRB during cell differentiation by taking advantage of site-specific phospho-serine/threonine antibodies. We first used the human histiocytic lymphoma cell line U-937 as a differentiation model since U-937 cells are induced to monocytic/macrophage-like differentiation by phorbol esters (Fig. S2A, B, C), ATRA, 1-α-25-dihyroxycholecalciferol (vitamin D3), gamma interferon, or tumor necrosis factor [25]–[27]. As shown in Fig. 1A, when U-937 cells were treated with TPA, most of the serine/threonine residues tested were dephosphorylated as the cells were differentiated, and this was reflected by the increase in reactivity of the anti-underphosphorylated pRB antibody (Fig. 1A, under-P) and the accumulation of faster migrating hypophosphorylated pRB. However, only Ser612 remained phosphorylated during differentiation. The phosphorylation level of Ser612 peaked between 24 and 48 h after the induction of differentiation. The asterisks in the panels of the anti-phspho-Thr356 immunoblot denote non-specific bands. We further examined other cell differentiation models, namely the hemin-induced erythroid differentiation model [28] and the TPA-induced megakaryocytic differentiation model [29], in human chronic myelogenous leukemia cell line K562 [30] (Fig. 1B, C and Fig. S2D, E). Similarly, most serine/threonine residues tested were dephosphorylated as K562 cells were differentiated by hemin or TPA, but only Ser612 in pRB remained phosphorylated by both stimuli and it remained phosphorylated at least 96 h after the induction of differentiation. We subsequently examined a different differentiation model in a human neuroblastoma cell line. Treatment with ATRA results in neural differentiation in SH-SY5Y cells (Fig. S2F) [31]. As shown in Fig. 1D, most of the serine/threonine residues in pRB were dephosphorylated when the cells were differentiated. As we expected, Ser612 remained phosphorylated during ATRA treatment. It is interesting to note that pRB phosphorylated at Ser612 was found even in the faster migrating hypophosphorylated form of pRB in all differentiation models tested. These results suggest that phosphorylation of Ser612 in pRB is regulated by a mechanism different from that of other serine/threonine residues in differentiated cells. It is conceivable that the mechanism is conserved in several cell differentiation models and indicates that phosphorylation of Ser612 endows pRB with a specific function.

### Anti-phospho-Ser612 Antibodies Recognize pRB only When it is Phosphorylated at Ser612

**Figure 2:**
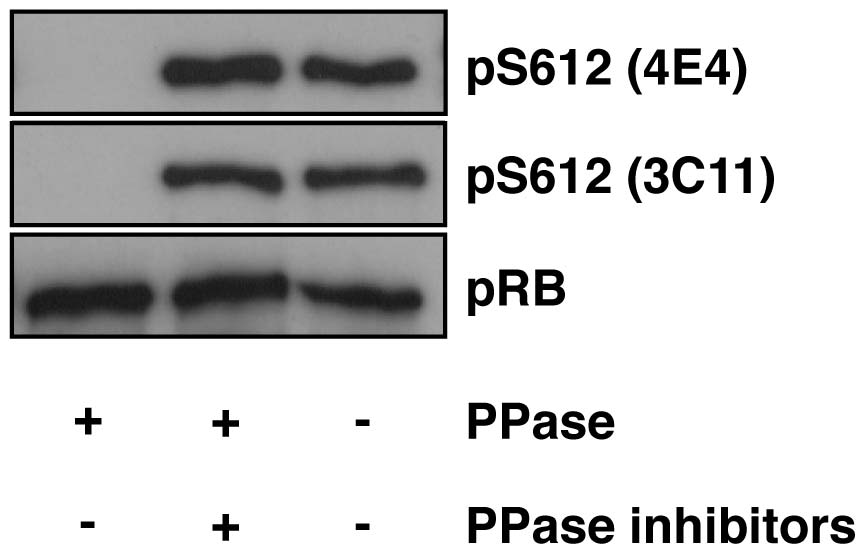
Anti-phospho-Ser612 antibodies recognize pRB only when it is phosphorylated at Ser612. Recombinant pRB was synthesized as described in the Materials and Methods section. pRB was incubated with or without λ phosphatase (PPase) in the absence or presence of a phosphatase inhibitor cocktail. The reaction was stopped by adding the Laemmli sample buffer and then the sample was analyzed by immunoblotting with the indicated antibodies.

**Figure 3:**
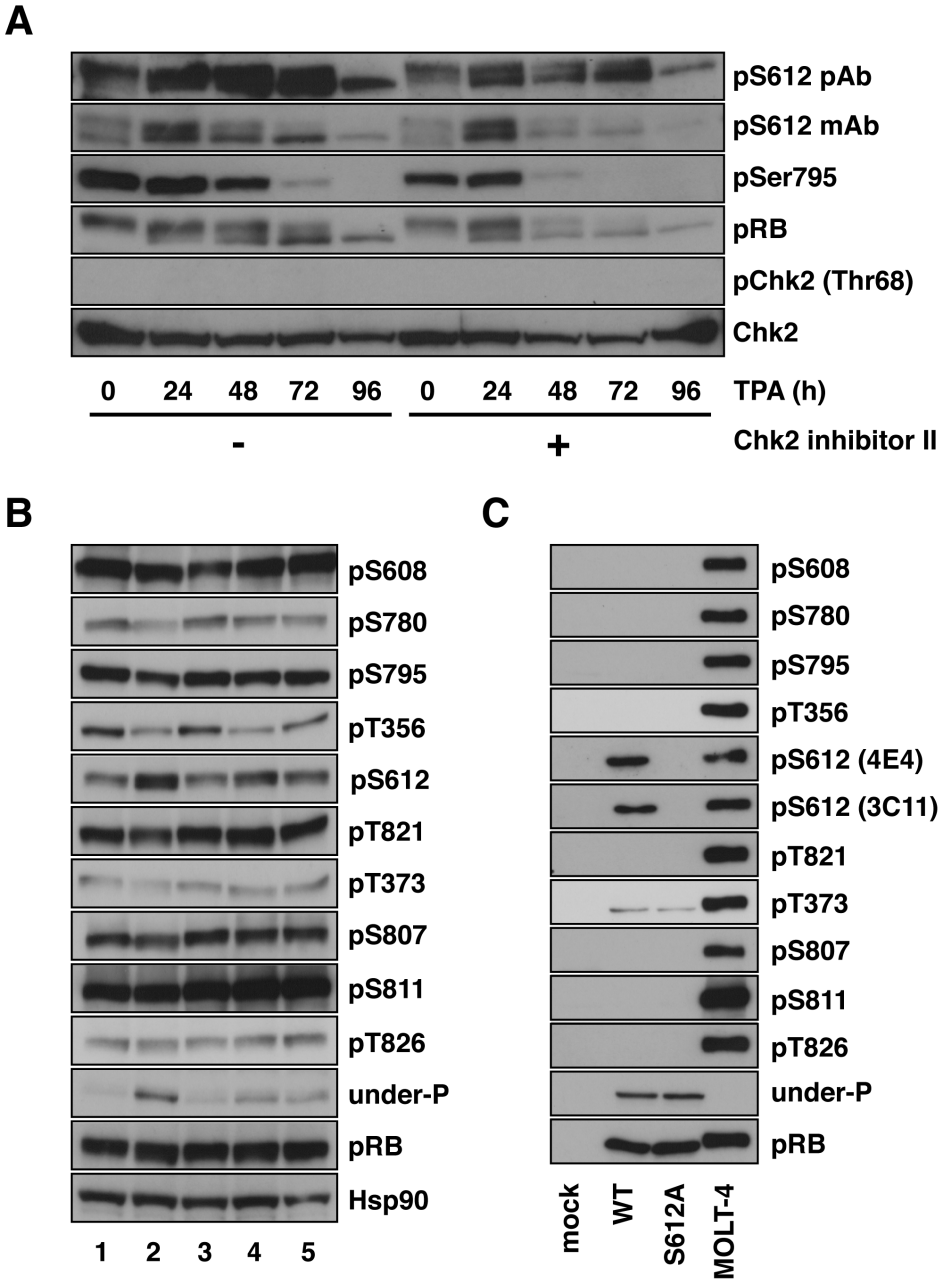
Phosphorylation of pRB at Ser612 is mediated by an unknown kinase in particular contexts. (A) U-937 cells were pretreated with 10 µM of the Chk2 inhibitor II for 24 h, and the cells were then treated with 20 nM TPA. After the indicated periods, the cells were harvested for immunoblotting with the indicated antibodies. (B) MOLT-4 cells were treated with 0.1% DMSO (lane 1), 1 µM GW8510 (lane 2), 10 µM Chk2 inhibitor II (lane 3), 1 µM SB218078 (lane 4) or 50 µM PD98059 (lane 5) for 6 h, then the cells were harvested for immunoblotting with the indicated antibodies. All compounds were dissolved in DMSO as a 1000 times stock solution. (C) SAOS-2 cells were transfected with empty (mock), wild-type RB (WT), or Ser612Ala RB (S612A) expression vectors. Forty-eight hours after transfection, the cells were harvested for immunoblotting with the indicated antibodies. A cell extract prepared from MOLT-4 cells was also loaded as a control for the antibodies.

We next verified the validity of the anti-phospho-Ser612 antibodies used in this study to ensure their substrate specificity. Recombinant pRB produced by a wheat germ cell-free protein synthesis system was phosphorylated at Ser612 (Fig. 2). When pRB was incubated with λ phosphatase, anti-phospho-Ser612 antibodies no longer recognize pRB. The reactivity was maintained when the incubation was performed in the presence of phosphatase inhibitors. Furthermore, anti-phospho-Ser612 antibodies were not able to recognize pRB when Ser612 was substituted with Ala (Fig. 3C). These results demonstrate that the anti-phospho-Ser612 antibodies used in this study do not recognize pRB when Ser612 is dephosphorylated.

### An Unknown Kinase Phosphorylates pRB at Ser612 in Particular Contexts

Studies have shown that pRB can be phosphorylated by cyclin/CDK complexes [2]. In addition, we recently reported that pRB can be phosphorylated at Ser612 in response to DNA damaging stimuli by checkpoint kinase 2 (Chk2) [18]. Serine/threonine residues other than Ser612 in pRB are dephosphorylated (Fig. 1). In addition, cell cycle progression is arrested in the G0/G1 phase in differentiated cells. Thus, it is highly unlikely that cyclin/CDK complexes are activated in those cells. Therefore, we first monitored the effect of a Chk2 inhibitor on the phosphorylation of pRB at Ser612. As shown in Fig. 3A, treatment with the Chk2 inhibitor II induced a slight downregulation of the pRB protein level. On the other hand, the Chk2 inhibitor II did not affect TPA-induced pRB conversion from the slower migrating hyperphosphorylated form to the faster migrating hypophosphorylated form, as represented by the dephosphorylation of Ser795. Chk2 inhibitor II treatment did not suppress phosphorylation of Ser612 in any time points either. This was supported by data that Chk2 was not activated during differentiation assessed by phosphorylation of Chk2 at Thr68, which is a prerequisite for the subsequent activation step (Fig. 3A). Conversely, DNA damage-induced phosphorylation of Ser612 was inhibited by pretreatment with the Chk2 inhibitor II (Fig. S3). This result suggests that Chk2 is not responsible for phosphorylation of pRB at Ser612 in response to differentiation stimuli.

We further investigated the involvement of cyclin/CDK complexes and Chk2 in Ser612 phosphorylation in other environments. The exponentially growing acute lymphoblastic leukemia cell line, MOLT-4 cells, was cultured with or without a CDK2 inhibitor, GW8510 or Chk2 inhibitor II, for 6 h, and phosphorylation of pRB was evaluated by site-specific phospho-serine/threonine antibodies (Fig. 3B). Treatment with GW8510 resulted in the apparent or slight dephosphorylation of Thr356, Thr373, Ser780, Ser795, Ser807 and Thr821 residues, and increased the reactivity of the anti-underphosphorylated pRB antibody (Fig. 3B, lane 2). Surprisingly, phosphorylation of Ser612 was rather upregulated by GW8510 treatment. On the other hand, inhibition of Chk2 did not influence the phosphorylation status of all serine/threonine residues tested (Fig. 3B, lane 3). This is probably because the assay was performed under non-stressed conditions. Only phosphorylation of the Thr356 residue was downregulated by treatment with the Chk1 (SB218078) or MEK1 (PD98059) inhibitors (Fig. 3B, lanes 4 and 5).

The human osteosarcoma cell line SAOS-2 lacks full-length functional nuclear pRB [32]. The reintroduction of the full-length *RB* gene into SAOS-2 cells produces pRB migrating as a single sharp band consistent with pRB in the hypophosphorylated state, because of the vanishingly low cyclin/CDK activity [33]. Ectopic expression of cyclins in SAOS-2 cells can promote phosphorylation of pRB and inactivate pRB via protein phosphorylation [33]. Indeed, most of the serine/threonine residues tested in introduced pRB were dephosphorylated in SAOS-2 cells and this was reflected by the reactivity of the anti-underphosphorylated pRB antibody (Fig. 3C). Nevertheless, only Ser612 was phosphorylated in SAOS-2 cells. This result once more suggests that Ser612 was phosphorylated by a kinase other than cyclin/CDK complexes, which phosphorylate most serine/threonine residues in pRB in SAOS-2 cells. Taken together, there must be an unknown Ser612-specific kinase other than CDKs and Chk2 in at least particular cellular contexts.

### pRB Phosphorylated at Ser612 Associates with the Nuclear Structure in Differentiated Cells

**Figure 4:**
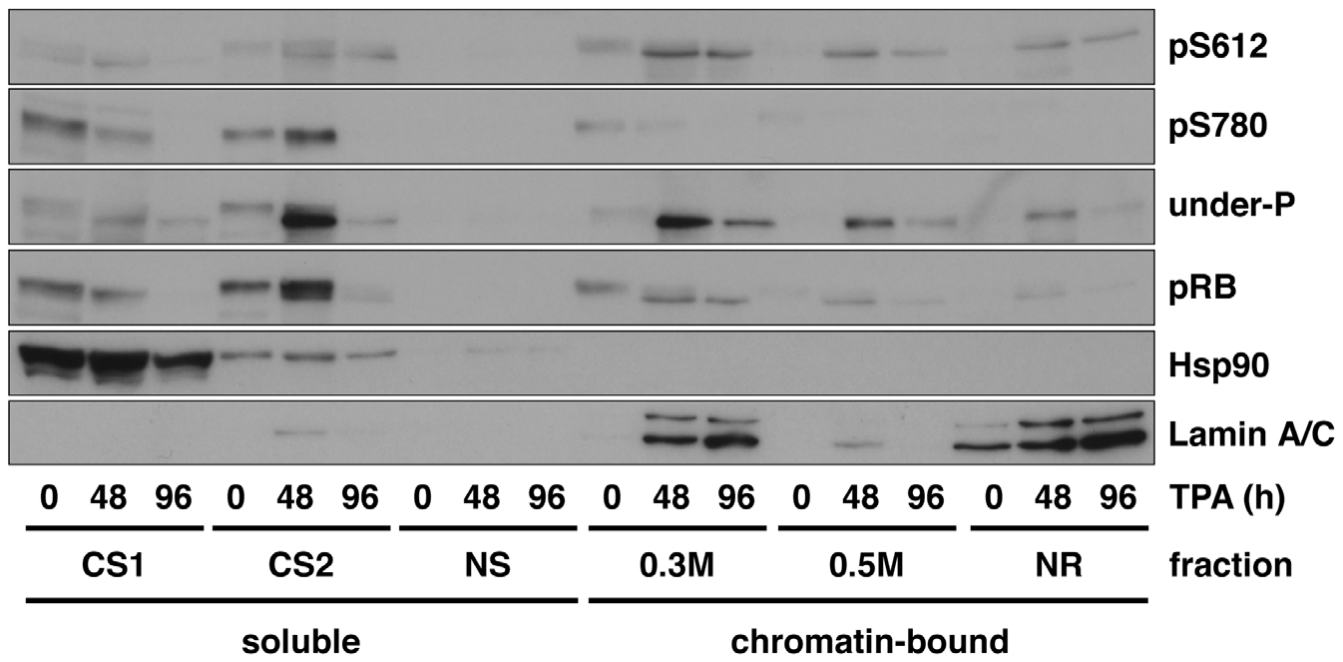
pRB phosphorylated at Ser612 tightly associates with chromatin and the nuclear structure in differentiated cells. U-937 cells were treated with 20 nM TPA for the indicated periods and the cells were harvested. The cells were fractionated as described in the Materials and Methods section. The extracts were analyzed by immunoblotting with the indicated antibodies.

Differentiation is typically coupled with cell cycle exit and hence is usually accompanied by dephosphorylation of pRB. It has been reported that hypophosphorylated pRB tightly associates with chromatin and the nuclear matrix in cell cycle exiting cells and that hyperphosphorylated pRB is found in the low-salt soluble fractions, which is interpreted to represent the cytoplasmic contents [15]. Therefore, we next examined in which fraction Ser612 phosphorylated pRB is distributed. As shown in Fig. 4, Ser612-phosphorylated pRB was found in chromatin and nuclear matrix fractions in response to induction of differentiation, whereas Ser780 phosphorylated pRB was mainly distributed in chromatin-unbound fractions in undifferentiated cells. The distribution of anti-phospho-Ser612 antibody-reactive pRB was quite similar to that of anti-underphosphorylated pRB antibody-reactive pRB (Fig. 4, under-P). These results demonstrate that pRB can associate with nuclear structures even when the Ser612 residue is phosphorylated in differentiated cells. We then examined the subcellular distribution of the Ser612 mutant pRB. Fig. S4 and Fig. S5 show that Ser612Ala pRB and Ser612Asp pRB distributed similarly to wild-type pRB, and that these proteins were predominantly localized in the nucleus.

### pRB Phosphorylated at Ser612 Can Bind to E2F-1

**Figure 5:**
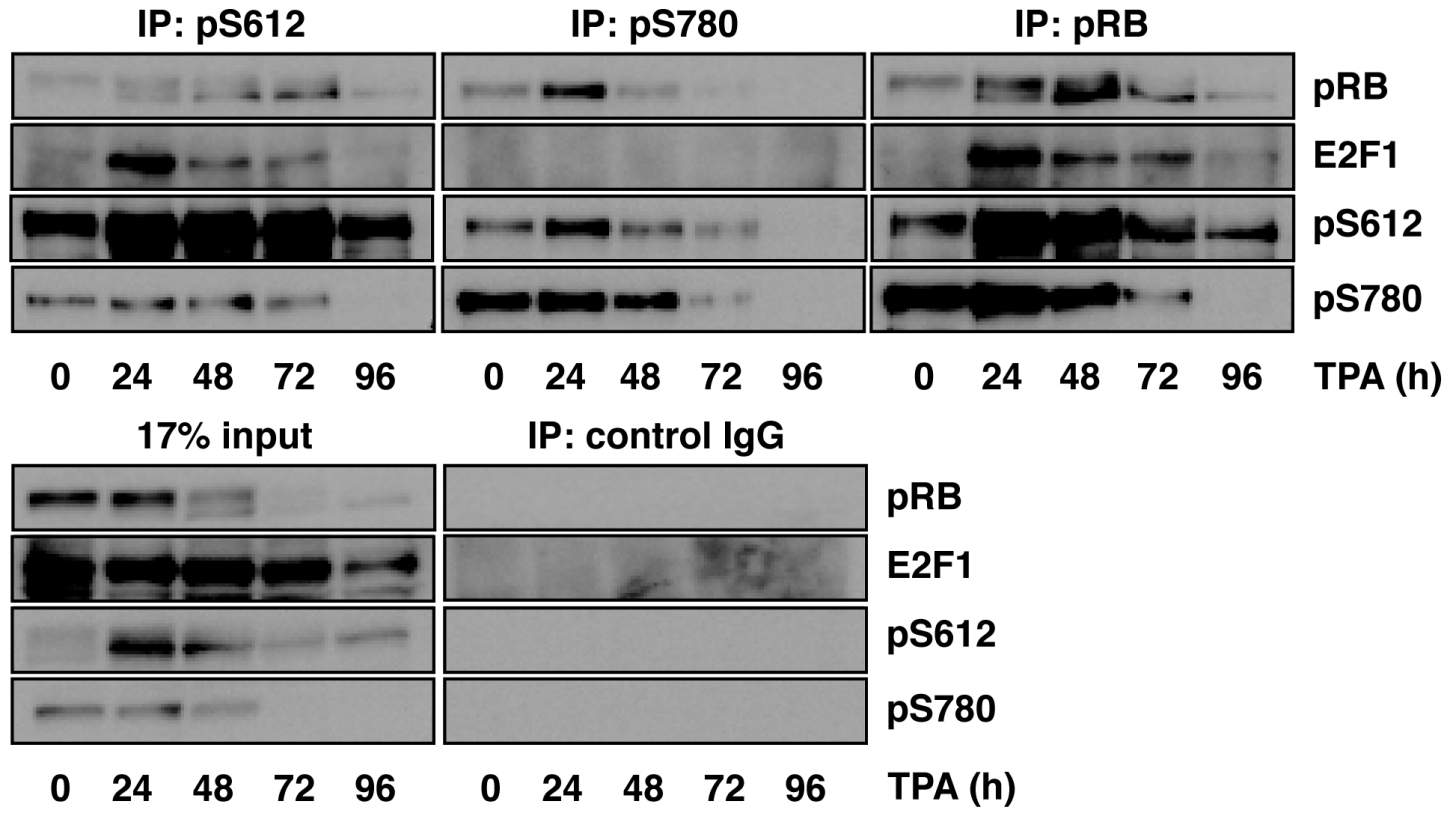
pRB phosphorylated at Ser612 does not release E2F-1. U-937 cells were treated with 20 nM TPA for the indicated periods and the cells were harvested. Cell extracts were prepared as described in the Materials and Methods section. Immunoprecipitation and immunoblotting was performed with the indicated antibodies.

The growth inhibitory properties of pRB are primarily mediated by its interaction with the E2F family of proteins. Inactivation of pRB by phosphorylation leads to the dissociation and activation of E2F, allowing the expression of several genes required for cell cycle progression and S phase entry [34]. It has been shown that Cdk4-Cyclin D1, but not Cdk2-Cyclin E specifically phosphorylates Ser780 in pRB, and that pRB phosphorylated at Ser780 does not bind to E2F-1 [19]. Thus, we next investigated whether pRB phosphorylated at Ser612 binds to E2F-1 in differentiated cells. E2F-1 associating with pRB was markedly increased at 24 h after TPA treatment and then gradually decreased as the cells underwent differentiation (Fig. 5, IP: pRB) parallel with a decrease in the intracellular pRB level (Fig. 5, 17% input). Consistent with a previous report [19], E2F-1 was absolutely absent in the anti-phospho-Ser780 immune complex (Fig. 5, IP: pSer780). On the other hand, E2F-1 was co-purified with Ser612-phosphorylated pRB (Fig. 5, IP: pSer612). In addition, we found that the pRB mutant in which Ser612 was substituted with Ala also associated with endogenous E2F-1 in RB-deficient C33A cells (Fig. S6). Phosphorylated Ser612 was found in the anti-phospho-Ser780 immune complex and reciprocally phosphorylated Ser780 was found in the anti-phospho-Ser612 immune complex. This observation suggests that phosphorylation of Ser612 coincides with the phosphorylation of other serine/threonine residues in pRB (Fig. 5, IP: pSer612 and IP: pSer780). Moreover, the results indicate that pRB phosphorylated at Ser612 does not release E2F-1, in other words, pRB associating with E2F-1 can be phosphorylated at Ser612.

### Phosphorylation of pRB at Ser612 is Involved in Differentiation

**Figure 6:**
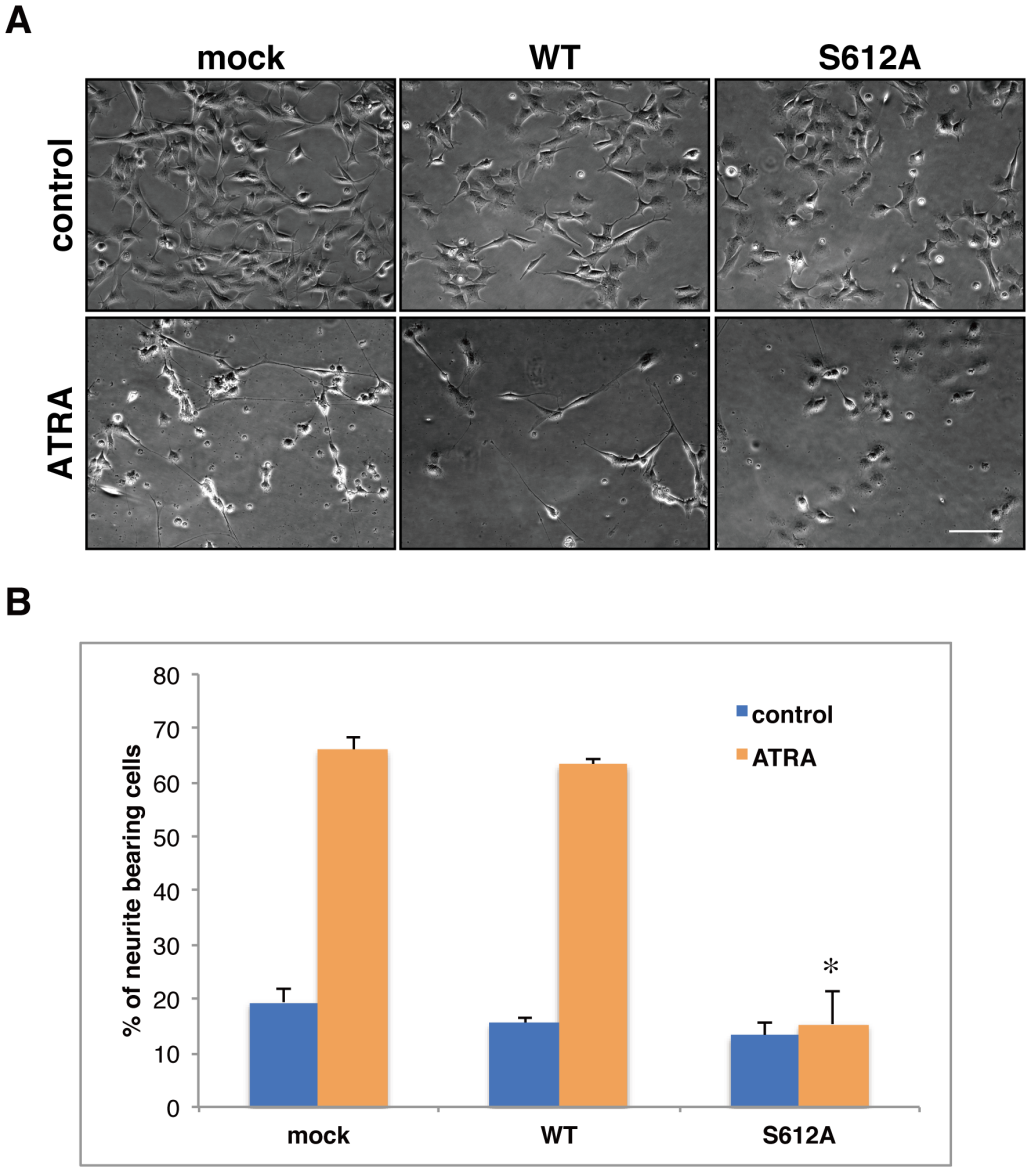
Expression of the pRB Ser612Ala mutant impairs the ability of the differentiation in ATRA-treated SH-SY5Y cells. (A) SH-SY5Y cells were transfected with empty (mock), wild-type RB (WT), or Ser612Ala RB (S612A) expression vectors. After 24 h, the cells were cultured in the presence or absence of 10 µM ATRA in 200 µg/ml hygromycin B containing medium for 14 d. Bar: 100 µm. (B) Neurite bearing cells were counted and the percentage of differentiated cells per field was calculated. Values are the means ± standard deviation of 3 independent fields. An asterisk represents a statistically significant difference from the values at ATRA-treated mock cells and ATRA-treated WT cells (*p*<0.001, *t*-test).

Finally, we examined the biological importance of the phosphorylation of Ser612 in differentiation. To test this, we transfected with the wild-type RB and Ser612Ala mutant RB expression vectors into SH-SY5Y cells. After transfection, the cells were simultaneously treated with ATRA and hygromycin B to induce differentiation and to select transfected cells. As shown in Fig. 6, wild-type RB-transfected cells were differentiated as well as the mock transfection. However, expression of the Ser612Ala mutant pRB significantly impaired the induction of differentiation. This result indicates that phosphorylation of Ser612 is actually involved in differentiation in SH-SY5Y cells.

## Discussion

In this report, we have shown that phosphorylation of Ser612 in pRB is regulated in a different manner from other serine/threonine residues. pRB is phosphorylated by cyclin/CDK complexes in response to growth stimuli; however, most studies describing pRB phosphorylation have focused on the bulk phosphorylation of pRB. In this study, we have made the first detailed examination of phosphorylation of each serine/threonine residue in pRB by taking advantage of site-specific phospho-pRB antibodies. In addition to the observations described above, we have also noticed that Ser612 remained phosphorylated even in the serum starved human glioblastoma cell line T98G and the human normal fibroblast cell line WI38, where other serine/threonine residues were dephosphorylated (Hattori et al., unpublished data). It has been reported that Ser612 can be phosphorylated by cyclin/CDK complexes [2]. However, these kinases are not responsible for the phosphorylation of Ser612 in quiescent cells such as differentiated cells, pRB-reintroduced SAOS-2 cells and serum starved cells, because the activity of cyclin/CDK complexes are very low in quiescent cells. Although Chk2 has been also reported to be a Ser612 kinase in cells exposed to DNA damaging stimuli [18], it does not appear to phosphorylate Ser612 in differentiated cells either. This is presumably because Chk2 is not activated in response to differentiation signals. Moreover, pretreatment with the Chk2 inhibitor does not block Ser612 phosphorylation in steady state cells and in differentiated cells. Consequently, there must be another kinase that phosphorylates pRB in particular cellular contexts.

Unlike phosphorylation of most serine/threonine residues in pRB, which promotes dissociation of the pRB-E2F-1 complex, pRB phosphorylated at Ser612 associates with E2F-1. Although hyperphosphorylated pRB is found in the soluble fraction [15], Ser612-phosphorylated pRB still associates with chromatin and the nuclear matrix as tightly as hypophosphorylated pRB does. The phosphorylation at Ser612 may provide pRB with a specific biological function that is distinct from any other known functions such as the release of E2F-1 and cell cycle regulation mediated by phosphorylation of other serine/threonine residues. Indeed, the forced expression of the Ser612Ala mutant pRB impaired the induction of differentiation in SH-SY5Y cells. Further studies are required to uncover the regulation mechanism mediated by Ser612 phosphorylation. We have isolated some proteins that associate with Ser612Ala pRB with higher affinity than wild-type pRB by mass spectrometry analysis of pRB-interacting proteins. These proteins may regulate cell differentiation by recognizing the phosphorylation status of pRB at Ser612.

It has been reported that reintroduction of pRB into SAOS-2 cells that lack full-length *RB* induces osteogenic and senescence-like differentiation [35]. We then investigated whether the expression of wild-type or a Ser612Ala mutant pRB into SAOS-2 cells gave rise to distinct phenotypes. Unexpectedly, reintroduction of the Ser612Ala mutant pRB into SAOS-2 cells could induce a senescence phenotype at the same level as wild-type pRB (Fig. S1B), even though Ser612 is the only residue that can be phosphorylated in this cell line (Fig. 3C). It seems that phosphorylation of the Ser612 residue is not involved in pRB-induced senescence-like morphological changes in SAOS-2 cells.

To explore the significance of the phosphorylation of Ser612 further, we tried to identify changes in gene expression between SAOS-2 cells expressing wild-type pRB and those expressing the Ser612Ala mutant pRB by microarray analysis. Surprisingly, there was no difference in gene expression between wild-type and Ser612Ala pRB introduced SAOS-2 cells (Hattori et al., unpublished data). This suggests that phosphorylation of Ser612 does not function in at least the SAOS-2 cell model.

It is important to ascertain whether the phosphorylation of pRB at Ser612 occurs in differentiated tissues *in vivo*. We tried to test this experimentally using mouse tissue samples or blood samples. Unfortunately, the anti-phospho-Ser612 antibodies employed (monoclonal antibody clones 3C11 and 4E4 and the polyclonal antibody) did not recognize the corresponding antigen of other species, including mouse. As far as we know, there is no anti-phospho-Ser612 antibody that recognizes an antigen from another species other than from human origin. This is probably because the surrounding amino acid residues differ in other species (Fig. S7). We think that Ser612 is phosphorylated in differentiated normal tissues as phosphorylation of this residue is also observed in normal human fibroblasts, which show cell cycle arrest in the G0 phase as described above. Experiments using human tissue samples are required in the future.

In Fig. 5, co-precipitated pRB was increased in anti-pSer780 immune complex at 24 h after TPA treatment. Phosphorylation of pRB at Ser612 was markedly increased at this time point. pRB phosphorylated at Ser612 might be precipitated with anti-pSer780 antibody with higher affinity than Ser612-unphosphorylated pRB because of the conformational change of this protein by the phosphorylation.

There are reports describing phosphorylation of pRB at Ser567, Ser780, Ser795, Ser811 and Tyr805 by distinct kinases including p38, ERK1/2, Abl and AMP-activated protein kinase [5]–[11]. However, these reports did not mention phosphorylation of Ser612 in pRB. The pRB phosphorylation described in those reports inactivates pRB and dissociates E2F or destabilizes pRB. That suggests that Ser612 phosphorylation, which does not cause the dissociation of E2F-1, has a unique role. Further research is required to understand the biological significance of phosphorylation of pRB at Ser612 in differentiated cells.

## Supporting Information

Figure S1 **Induction of osteogenic and senescence-like differentiation in pRB-inducible SAOS-2 cells.** (A) pRB-inducible SAOS-2 cell clones described in the Material and Method section were treated with indicated concentration of doxycycline (Dox) for 24 h and then the cells were harvested for immunoblotting with the indicated antibodies. (B) SAOS-2 cell clones in which pRB is induced by doxycycline (Dox) treatment were cultured in the presence of indicated concentrations of Dox for 14 d. Osteogenic and senescence-like differentiated cells were stained with the senescence β-galactosidase staining kit. Bar: 100 µm.
(TIF)

Figure S2 **Induction of differentiation in U937, K562 and SH-SY5Y cells.** (A–C) Monocytic/macrophage-like differentiation of U937 cells. The cells were treated with 20 nM TPA for 96 h. (A) Macrophage-like morphological change in TPA-treated cells. (B) Nitroblue tetrazolium (NBT) reducing assay. TPA-treated cells reduced NBT and resulted in blue formazan staining. (C) Expression of CD11b (integrin αM) in TPA-treated cells was assessed by flow cytometric analysis with V450-conjugated anti-CD11b antibody (BD Biosciences). (D) Induction of erythroid differentiation in K562 cells. The cells were treated with 30 µM hemin. After 96 h, the benzidine staining assay was performed. Hemin-treated cells showed blue in color. (E) Megakaryocytic differentiation in K562 cells. The cells were treated with 10 nM TPA and then the morphological change was observed. (F) Neural morphological change in ATRA-treated SH-SY5Y cells. The cells were treated with 10 µM ATRA for 14 d. ATRA promoted neurite outgrowth.
(TIF)

Figure S3 **Chk2-dependent phosphorylation of pRB at Ser612 in DNA-damaged cells.** MOLT-4 cells were pretreated with 10 µM of the Chk2 inhibitor II for 1 h and the cells were then treated with 20 µg/ml etoposide. After the indicated periods, the cells were harvested for immunoblotting with the indicated antibodies.
(TIF)

Figure S4 **Subcellular distributions of Ser612 mutant pRB are identical to that of wild-type pRB.** U2OS cells were transfected with Myc-tagged wild-type RB (WT), Myc-tagged Ser612Ala RB (S612A), or Myc-tagged Ser612Asp RB (S612D) expression vectors. Two days after transfection, the cells were fractionated as described in the Materials and Methods section. The extracts were analyzed by immunoblotting with the indicated antibodies.
(TIF)

Figure S5 **The Ser612 mutant pRBs and the wild-type protein predominantly localize in the nucleus.** U2OS cells were transfected with Myc-tagged wild-type RB (WT), Myc-tagged Ser612Ala RB (S612A), or Myc-tagged Ser612Asp RB (S612D) expression vectors. After 48 h, the cells were fixed and stained with anti-Myc (pRB, green) and DAPI (nuclei, blue).
(TIF)

Figure S6 **pRB Ser612Ala mutant can bind to E2F-1.** C33A cells (*RB* deficient human cervical carcinoma) were transfected with empty, Myc-tagged wild-type RB (WT), or Myc-tagged Ser612Ala RB (S612A) expression vector. The cell lysates were subjected to immunoprecipitation with anti-Myc antibody-conjugated agarose. The precipitates were analyzed by Western blotting with anti-E2F1 and anti-pRB antibodies.
(TIF)

Figure S7 **Alignment of pRB sequences adjacent to Ser612 from human, rat and mouse.** Residues juxtaposition of human Ser612 (red in color) are not essentially identical between species.
(TIF)
