# Supplementary figures and images for: Distinct and Site-Specific Phosphorylation of the Retinoblastoma Protein at Serine 612 in Differentiated Cells
Source: PLoS One. 2014 Jan 21;9(1):e86709. doi: 10.1371/journal.pone.0086709 (PMC3897739; doi:10.1371/journal.pone.0086709)

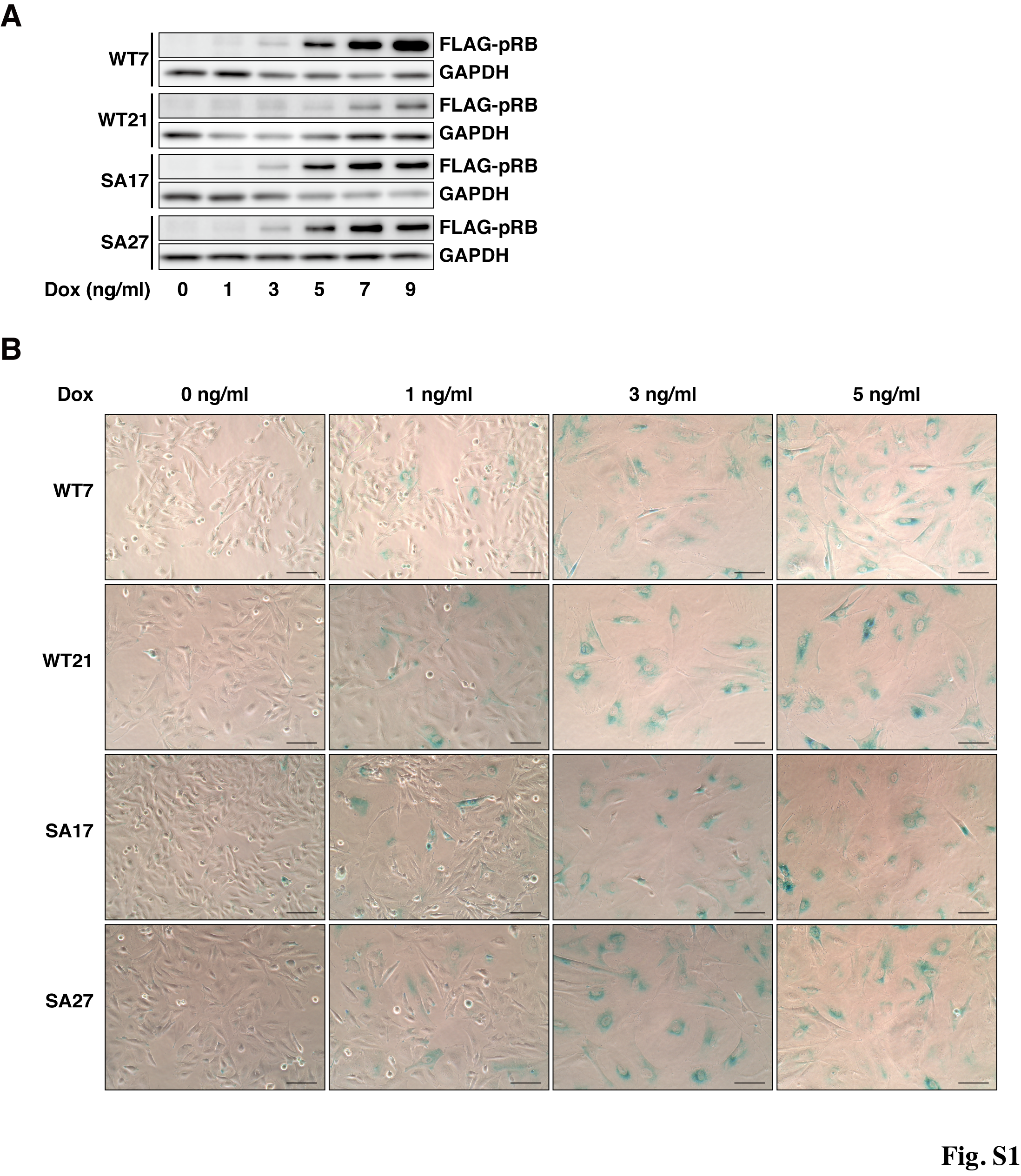

Supplement: Figure S1 — Induction of osteogenic and senescence-like differentiation in pRB-inducible SAOS-2 cells. (A) pRB-inducible SAOS-2 cell clones described in the Material and Method section were treated with indicated concentration of doxycycline (Dox) for 24 h and then the cells were harvested for immunoblotting with the indicated antibodies. (B) SAOS-2 cell clones in which pRB is induced by doxycycline (Dox) treatment were cultured in the presence of indicated concentrations of Dox for 14 d. Osteogenic and senescence-like differentiated cells were stained with the senescence β-galactosidase staining kit. Bar: 100 µm. (TIF) [file pone.0086709.s001.tif]

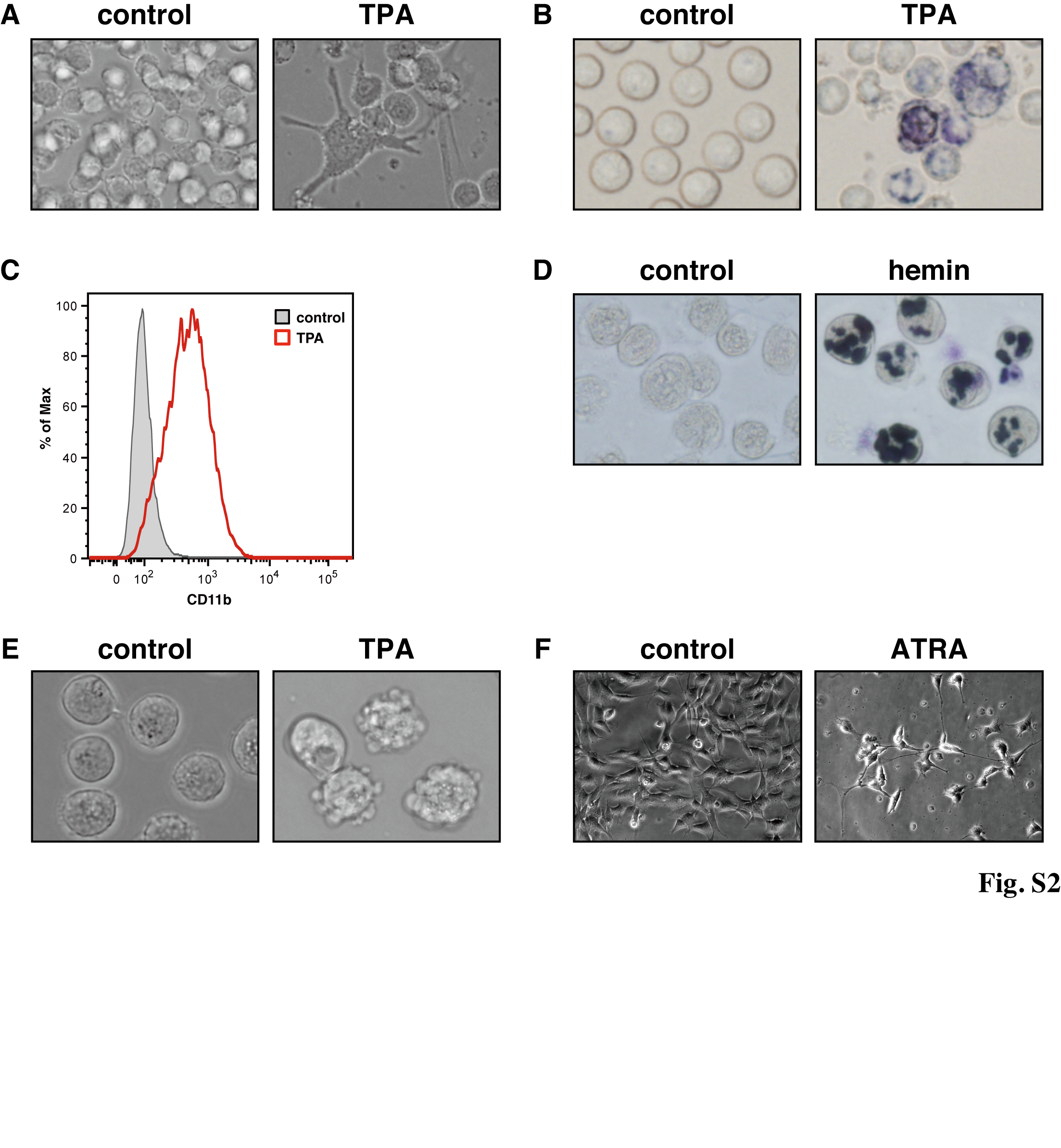

Supplement: Figure S2 — Induction of differentiation in U937, K562 and SH-SY5Y cells. (A–C) Monocytic/macrophage-like differentiation of U937 cells. The cells were treated with 20 nM TPA for 96 h. (A) Macrophage-like morphological change in TPA-treated cells. (B) Nitroblue tetrazolium (NBT) reducing assay. TPA-treated cells reduced NBT and resulted in blue formazan staining. (C) Expression of CD11b (integrin αM) in TPA-treated cells was assessed by flow cytometric analysis with V450-conjugated anti-CD11b antibody (BD Biosciences). (D) Induction of erythroid differentiation in K562 cells. The cells were treated with 30 µM hemin. After 96 h, the benzidine staining assay was performed. Hemin-treated cells showed blue in color. (E) Megakaryocytic differentiation in K562 cells. The cells were treated with 10 nM TPA and then the morphological change was observed. (F) Neural morphological change in ATRA-treated SH-SY5Y cells. The cells were treated with 10 µM ATRA for 14 d. ATRA promoted neurite outgrowth. (TIF) [file pone.0086709.s002.tif]

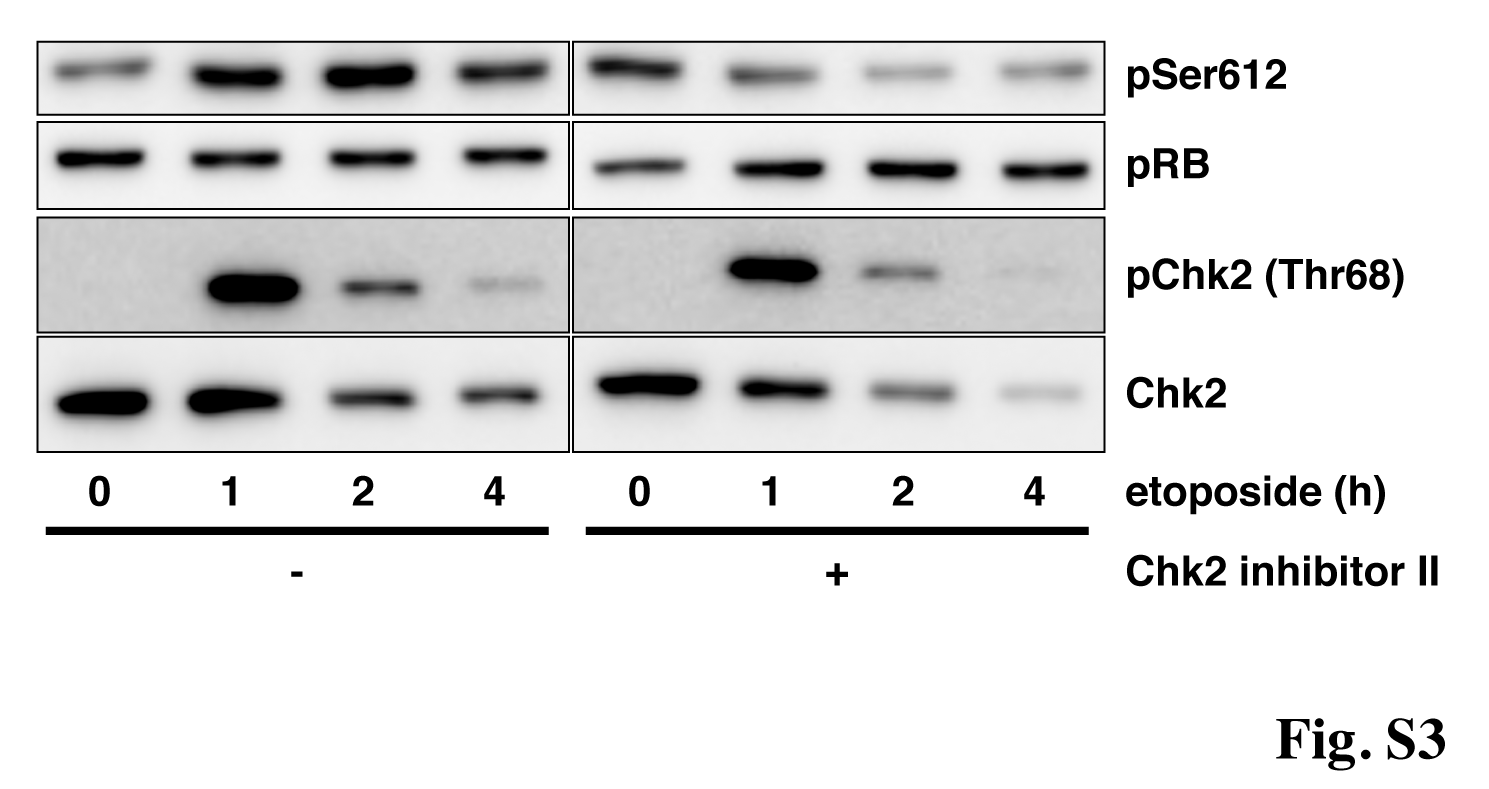

Supplement: Figure S3 — Chk2-dependent phosphorylation of pRB at Ser612 in DNA-damaged cells. MOLT-4 cells were pretreated with 10 µM of the Chk2 inhibitor II for 1 h and the cells were then treated with 20 µg/ml etoposide. After the indicated periods, the cells were harvested for immunoblotting with the indicated antibodies. (TIF) [file pone.0086709.s003.tif]

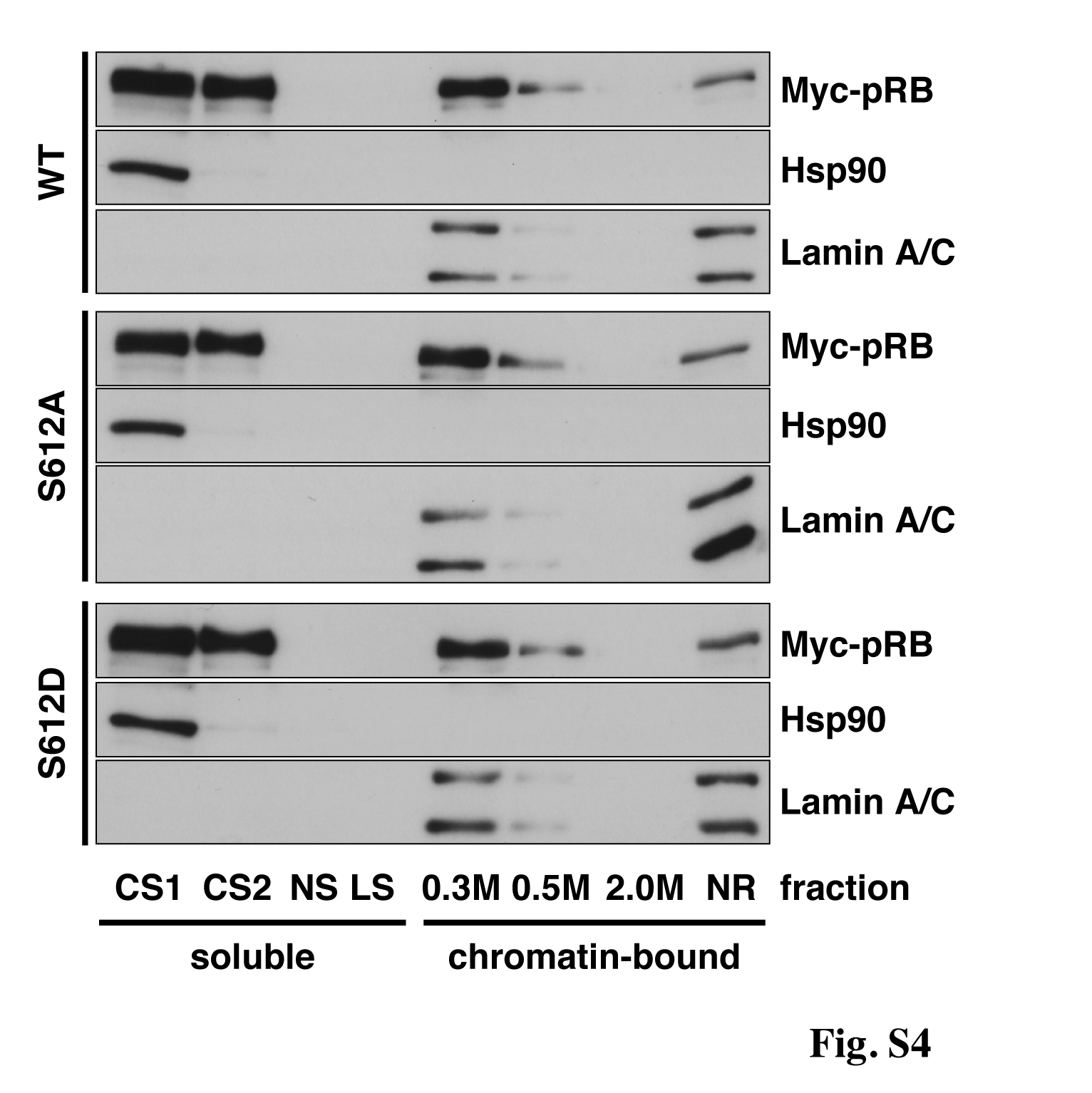

Supplement: Figure S4 — Subcellular distributions of Ser612 mutant pRB are identical to that of wild-type pRB. U2OS cells were transfected with Myc-tagged wild-type RB (WT), Myc-tagged Ser612Ala RB (S612A), or Myc-tagged Ser612Asp RB (S612D) expression vectors. Two days after transfection, the cells were fractionated as described in the Materials and Methods section. The extracts were analyzed by immunoblotting with the indicated antibodies. (TIF) [file pone.0086709.s004.tif]

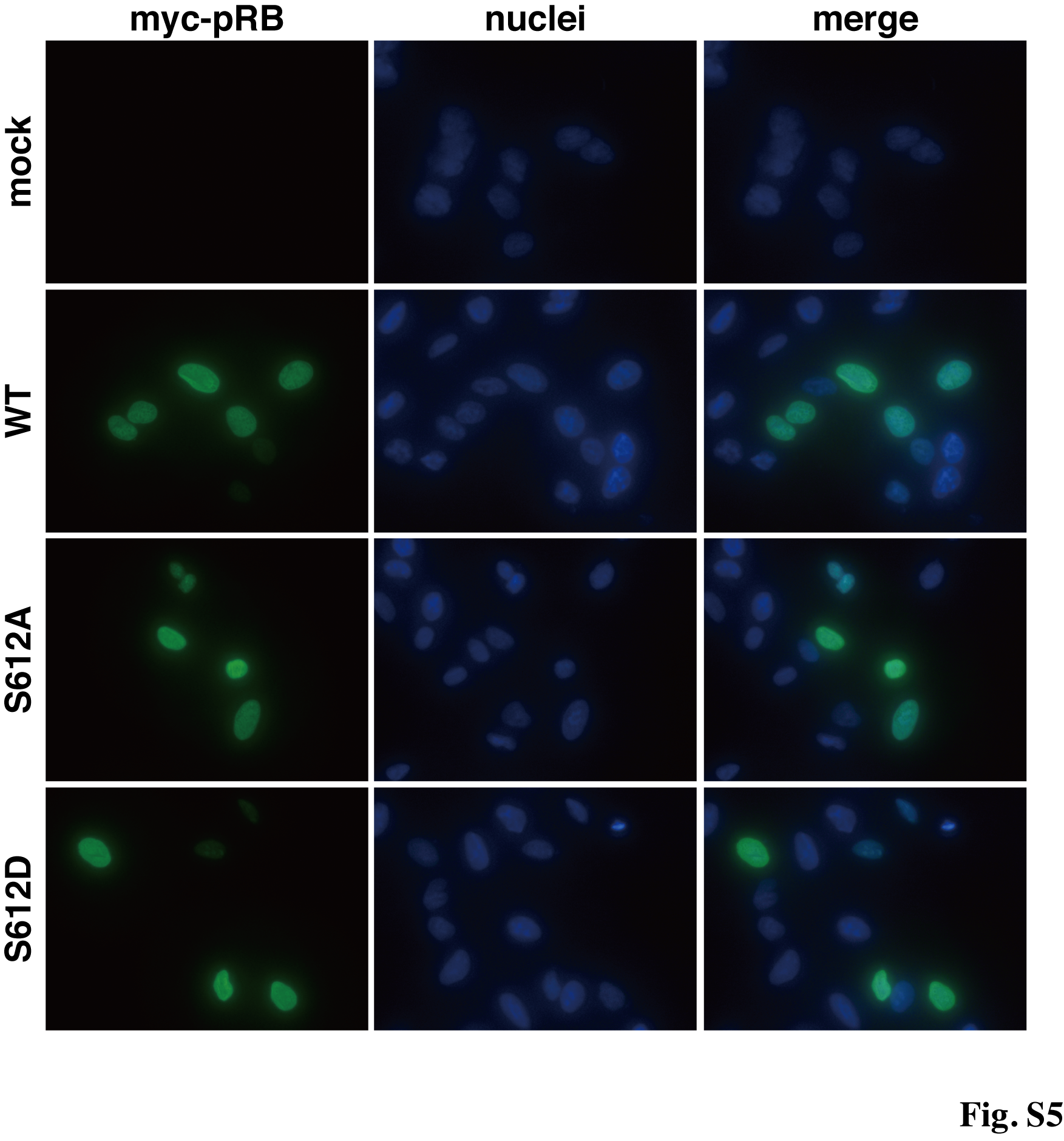

Supplement: Figure S5 — The Ser612 mutant pRBs and the wild-type protein predominantly localize in the nucleus. U2OS cells were transfected with Myc-tagged wild-type RB (WT), Myc-tagged Ser612Ala RB (S612A), or Myc-tagged Ser612Asp RB (S612D) expression vectors. After 48 h, the cells were fixed and stained with anti-Myc (pRB, green) and DAPI (nuclei, blue). (TIF) [file pone.0086709.s005.tif]

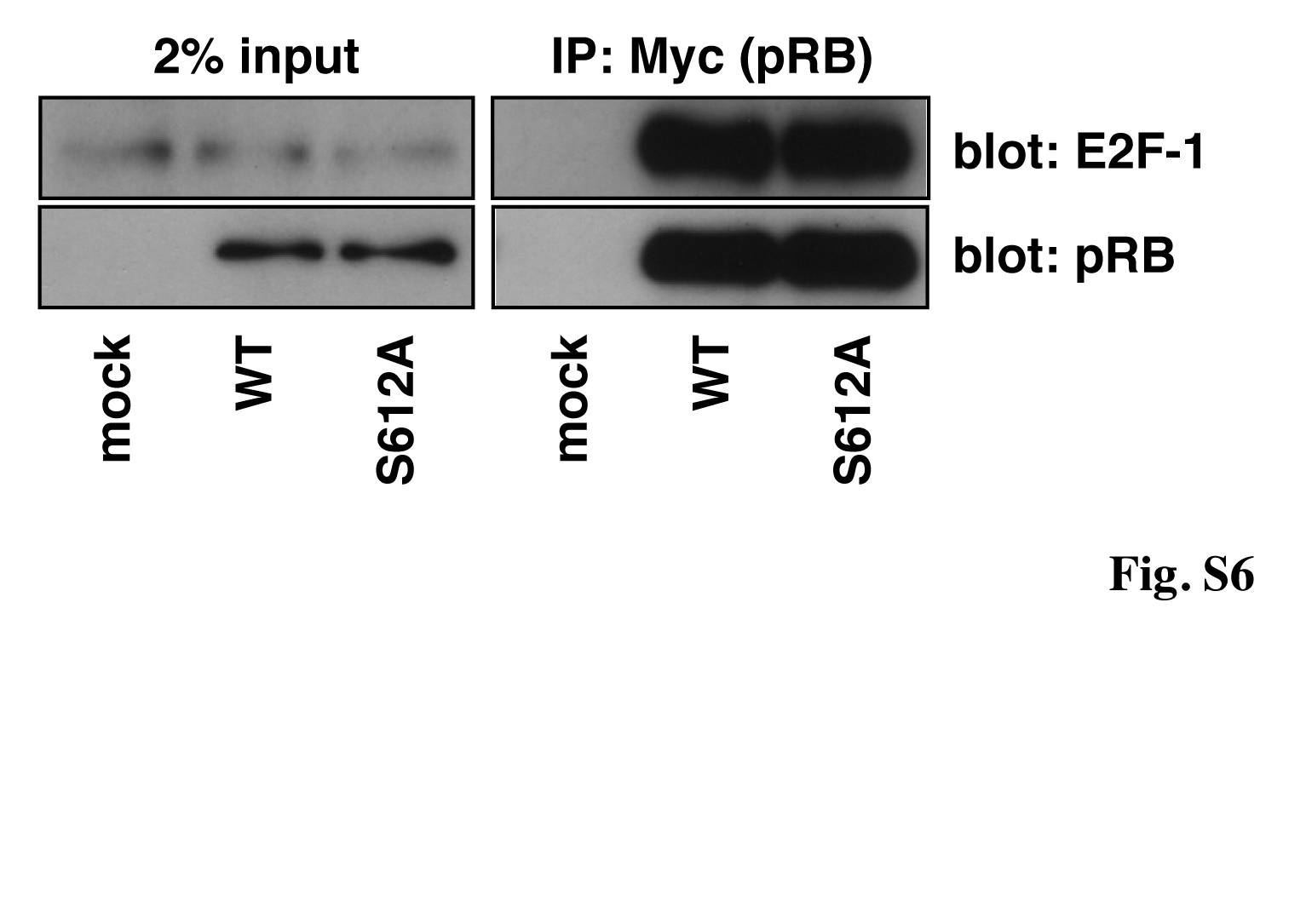

Supplement: Figure S6 — pRB Ser612Ala mutant can bind to E2F-1. C33A cells (RB deficient human cervical carcinoma) were transfected with empty, Myc-tagged wild-type RB (WT), or Myc-tagged Ser612Ala RB (S612A) expression vector. The cell lysates were subjected to immunoprecipitation with anti-Myc antibody-conjugated agarose. The precipitates were analyzed by Western blotting with anti-E2F1 and anti-pRB antibodies. (TIF) [file pone.0086709.s006.tif]

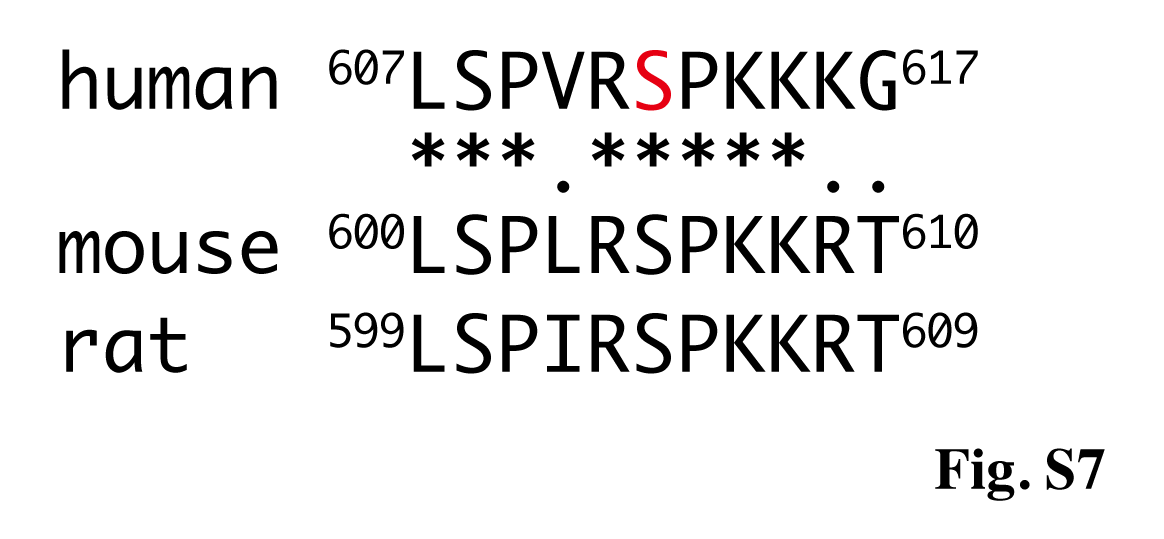

Supplement: Figure S7 — Alignment of pRB sequences adjacent to Ser612 from human, rat and mouse. Residues juxtaposition of human Ser612 (red in color) are not essentially identical between species. (TIF) [file pone.0086709.s007.tif]
